# Preservative Effect of Alga Flour Extract on Frozen Horse Mackerel (*Trachurus trachurus*) Lipids

**DOI:** 10.3390/foods13203265

**Published:** 2024-10-14

**Authors:** Miriam Martínez, Marcos Trigo, Santiago P. Aubourg, Alicia Rodríguez

**Affiliations:** 1 Department of Food Science and Technology, Instituto de Investigaciones Marinas (CSIC), 36208 Vigo, Spain; miriammartinezr03@gmail.com (M.M.); mtrigo@iim.csic.es (M.T.); 2 Department of Food Science and Chemical Technology, Faculty of Chemical and Pharmaceutical Sciences, University of Chile, Dr. Carlos Lorca Tobar 964, Santiago 8380494, Chile; arodrigm@uchile.cl

**Keywords:** red algae, flour, aqueous extract, frozen fish, glazing, fatty acids, oxidation, hydrolysis, trimethylamine, quality

## Abstract

The aim of this study was to investigate the preservative properties of alga *Gelidium* sp. flour when included in the glazing medium employed for the frozen storage (−18 °C) of horse mackerel (*Trachurus trachurus*). Different concentrations (low, medium, and high) of an aqueous extract were tested and compared to a control water-glazing condition. Quality changes (lipid oxidation and hydrolysis, fatty acid (FA) profile, and trimethylamine (TMA) formation) were determined after 3- and 6-month storage periods. A general quality loss (lipid oxidation with hydrolysis development and TMA formation) with the frozen storage period was detected in all samples. The presence of an alga flour (AF) extract in the glazing medium led to a lower (*p* < 0.05) TBARS and fluorescent compound formation and to higher (*p* < 0.05) polyene values in frozen fish. Furthermore, a preserving effect on free fatty acids was detected in AF-treated fish. On the contrary, the AF-glazing treatment did not affect (*p* > 0.05) the TMA formation and the total n3/total n6 FA ratio. In general, preservative effects were found to be higher in frozen fish corresponding to the medium concentration tested. Current results show the potential of *Gelidium* sp. flour as a natural source of preservative hydrophilic compounds for the quality enhancement of frozen horse mackerel.

## 1. Introduction

Frozen storage can mostly inhibit microbial development, but fish constituents may undergo different kinds of damage mechanisms such as formation of aggregates, protein insolubility, mechanical damage, and development of lipid oxidation and hydrolysis [1,2,3]. Consequently, different strategies and technologies have been investigated with the aim of enlarging shelf life during seafood storage under frozen conditions [4,5]. Among them, the employment of an ice layer on the frozen seafood, i.e., glazing, has attracted great attention [6,7]. It has been proved that glazing treatment of marine substrates prior to storage under frozen conditions can preserve the final product from oxidation and dehydration and lead to a general quality enhancement. Previous studies have proved remarkable effects of glazing by inhibiting the development of lipid hydrolysis [8] and oxidation [9,10] and microbial growth [11,12].

Marine species processing leads to the formation of great quantities of undesired waste and discarded materials (i.e., skin, viscera, blood, heads, bones, etc.). Such substrates constitute a remarkable source of environmental contamination, so that great efforts are mandatory for their recovery and to enhance their commercial value [13,14,15]. Notably, marine by-products can provide a valuable source of main constituents, as well as minor constituents such as chitin, collagen, enzymes, and pigments [16,17,18]. Consequently, great attention has been focused on accurate use of such compounds regarding the nutraceutical, pharmaceutical, and cosmeceutical industries [19,20].

Recently, the employment of seaweeds as food ingredients in Western countries has received increased attention as a relevant source of valuable molecules such as amino acids, vitamins, trace minerals, lipids, and dietary fiber [21,22]. Furthermore, there has been great interest in seaweeds because they contain a wide variety of minor constituents with potential antimicrobial [23,24] and antioxidant [25,26] activities. Among macroalgae, the red ones are greatly recognized for their commercial employment for phycocolloid (align, agar, carrageenan, and furcellaran) extraction [27,28]. As a result of this extraction process, large quantities of waste substrates such as alga flour (AF) are produced. Remarkably, recent research has shown this waste substrate to have valuable antimicrobial [29] and antioxidant [30] properties.

Horse mackerel is a semi-pelagic fish with a broad distribution in the northeast Atlantic Ocean, the Mediterranean Sea, and the Black Sea [31,32]. This species is well known for its great popularity worldwide and for its high nutritional value [33,34]. However, due to its high unsaturated fatty acid (FA) composition, horse mackerel is reported to undergo a fast quality loss during processing, especially regarding the lipid fraction [35,36].

The objective of this study was to analyze the preservative properties of an aqueous extract of red AF when added to the glazing system used for the fish freezing process. Different concentrations of an aqueous extract of *Gelidium* sp. were tested and compared to a control treatment (water-glazing condition) during horse mackerel (*Trachurus trachurus*) frozen storage. Quality changes (lipid oxidation and hydrolysis, FA profile, and trimethylamine (TMA) formation) were determined after frozen storage at −18 °C for 3 and 6 months.

## 2. Materials and Methods

All solvents and chemical reagents used in this study were of reagent grade (Merck, Darmstadt, Germany); otherwise, the source is mentioned.

### 2.1. Initial Alga Flour Composition

The initial flour was obtained from *Gelidium* sp. and was provided by Industrias Roko S. A. (Llanera, Asturias, Spain). AF is a brown-colored solid with a slight marine smell. Its proximate composition was analyzed according to the AOAC (Association of Official Agricultural Chemists) [37] procedure, and the analysis showed the following proximate composition (%): 7.75 ± 0.11 (moisture), 31.46 ± 1.27 (protein), 0.12 ± 0.02 (lipids), 6.54 ± 0.76 (ash), and 54.13 ± 1.81 (total carbohydrate).

The FA analysis of the *Gelidium* sp. flour was carried out in agreement with the methodology presented in Section 2.4. The following composition for individual FAs was obtained (g·100 g^−1^ total FAs): 6.69 ± 0.08 (C14:0), 0.96 ± 0.02 (C15:0), 66.85 ± 0.45 (C16:0), 2.43 ± 0.04 (C16:1n7), 0.60 ± 0.01 (C17:0), 3.41 ± 0.07 (C18:0), 7.82 ± 0.09 (C18:1n9), 1.88 ± 0.03 (C18:1n7), 0.62 ± 0.05 (C18:2n6), 0.47 ± 0.03 (C20:1n9), 0.15 ± 0.04 (C20:2n6), 2.91 ± 0.16 (C20:4n6), 0.20 ± 0.02 (C22:1n9), 1.90 ± 0.02 (C20:5n3), 1.10 ± 0.01 (C22:4n6), 0.15 ± 0.01 (C24:1n9), 0.38 ± 0.04 (C22:5n3), and 1.22 ± 0.27 (C22:6n3).

### 2.2. Preparation of Flour Extract and Glazing Systems

The aqueous extract of the AF was obtained in agreement with previous research [30]. AF (26 g) and distilled water (400 mL) were mixed and subsequently subjected to stirring (Vortex, Scientific Industries, Bohemia, NY, USA) for 30 s, sonication (J.P. Selecta, S.A., Barcelona, Spain) for 30 s, and centrifugation (Beckman Coulter ALLEGRA X12R, Brea, CA, USA) at 3500× *g* for 30 min at 4 °C, with the resulting supernatant being recovered. Then, the extraction process was repeated three more times. Finally, all four supernatants were pooled together and measured out to a volume of 2 L with distilled water. The resulting colorless and odorless solution corresponded to an alga flour concentration of 13 g·L^−1^.

Three glazing systems were prepared including the AF extract. For the extract, 150, 450, and 1350 mL of the above-mentioned solution were taken and diluted, respectively, to 6 L with distilled water. Thus, low-concentrated (L-AF; 0.325 g AF·L^−1^), medium-concentrated (M-AF; 0.975 g AF·L^−1^), and high-concentrated (H-AF; 2.925 g AF·L^−1^) glazing systems from AF were obtained, respectively. Additionally, a water-glazing condition (control batch, CTR) was considered as the control condition.

Concentrations of AF extract in the glazing solutions used in this experiment were chosen according to previous studies. Thus, a 0.2–5.0 g AF·L^−1^ range was tested. As a result, 3.0 g AF·L^−1^ showed to be the highest concentration that did not modify any of the sensory descriptors (i.e., odor, color, or taste) of the horse mackerel muscle. Consequently, this concentration was considered in the present study (i.e., 2.925 g·L^−1^), together with two less concentrated ones, to analyze the effect of AF concentration.

### 2.3. Initial Horse Mackerel, Freezing, Glazing, and Frozen Storage

Initial horse mackerel (54 individuals) (*T. trachurus*) were acquired at Vigo harbor (Galicia, Spain) and carried on ice to the laboratory. The length and weight of fish individuals ranged from 27 to 30 cm and from 115 to 140 g, respectively.

As a first step in the laboratory, six individuals were taken and considered as initial fish. These individuals were distributed into three different groups (two individuals per group) that were analyzed independently to carry out the statistical analysis (*n* = 3). The remaining fish individuals were distributed into four batches (12 individuals in each batch) and were subjected to the freezing process (48 h at −40 °C) (Sanyo Ultralow MDF-U53W, Moriguchi, Osaka, Japan).

After the freezing step, fish were immersed, respectively, in the previously described glazing conditions (i.e., CTR, L-AF, M-AF, and H-AF). Individuals were immersed (30 s at 4 °C), allowed to drain (15 s), packaged in polyethylene bags (two individuals per bag), and subjected to frozen storage at −18 °C (Radiber, Barcelona, Spain).

Fish individuals were taken for analysis after 3- and 6-month periods at −18 °C, respectively. At each sampling time and for each glazing condition, six individuals were taken and divided into three groups (two individuals per group) that were considered separately (*n* = 3). Frozen fish were analyzed after overnight thawing at 4 °C.

### 2.4. Analysis of Lipid Damage

Lipids of horse mackerel muscle were extracted by the Bligh and Dyer [38] method, which uses a chloroform/methanol (1/1, *v*/*v*) mixture. Total lipid value was calculated as g·kg^−1^ fish muscle.

The peroxide value (PV) was determined spectrophotometrically (520 nm) (Beckman Coulter DU 640 spectrophotometer, Brea, CA, USA) in the lipid extract according to the ferric thiocyanate method [39]. In this method, the ferric ions, produced by the oxidation of ferrous ions by peroxides, react with thiocyanate and lead to the formation of a pink-purple colored adduct. Results were expressed as meq. active oxygen·kg^−1^ lipids.

The thiobarbituric acid index (TBA-i) was determined spectrophotometrically (532 nm) in agreement with the Vyncke [40] method. This procedure is based on the reaction between a trichloroacetic acid extract of the fish muscle and a thiobarbituric acid (TBA) solution at high temperature (95–97 °C). For quantitative purposes, 1,1,3,3-tetraethoxy-propane was employed for preparing a standard curve. The presence of TBA reactive substances (TBARSs) was expressed as mg malondialdehyde·kg^−1^ fish muscle.

Fluorescent compound formation was analyzed with a Fluorimeter LS 45 (Perkin Elmer España; Tres Cantos, Madrid, Spain) at 327 nm/415 nm and 393 nm/463 nm in agreement with previous research [41]. According to this method, results were expressed as the fluorescence ratio (FR), which was determined in the aqueous phase resulting from the lipid extraction of the fish muscle.

The content of free fatty acids (FFAs) in fish muscle was analyzed spectrophotometrically (715 nm) in the lipid extract in agreement with the Lowry and Tinsley [42] procedure. This method is based on the formation of a colored complex between the acid group of FFAs and cupric acetate in the presence of pyridine at pH = 6.1. Results were expressed as g FFAs·kg^−1^ fish muscle.

### 2.5. Analysis of the FA Profile

Lipid extracts of horse mackerel muscle were converted into FA methyl esters (FAMEs) by employing acetyl chloride in methanol and then analyzed by gas chromatography (Perkin-Elmer 8700 chromatograph, Madrid, Spain) in agreement with previous research [43]. Thus, a fused silica capillary column SP-2330 (0.25 mm i.d. × 30 m, Supelco, Inc., Bellefonte, PA, USA) was used, and the following temperature program was applied: increased from 145 to 190 °C at 1.0 °C·min^−1^ and from 190 °C to 210 °C at 5.0 °C·min^−1^ and then held for 13.5 min at 210 °C. Nitrogen at 10 psig was used as the carrier gas, and detection was carried out with a flame ionization detector at 250 °C. A programmed temperature vaporizer injector was used in the split mode (150:1) and was heated from 45 to 275 °C at 15 °C·min^−1^.

Identification of FAME peaks was carried out by comparison of their retention times to those of standard mixtures (Qualmix Fish and Supelco 37 Component FAME Mix, Supelco, Inc., Bellefonte, PA, USA). Automatic integration of peak areas was carried out; quantification was achieved by employing C19:0 as the internal standard. The presence of each FA was determined as g·100 g^−1^ total FAs.

Results regarding FA groups (saturated FAs, STFAs; monounsaturated FAs, MUFAs; polyunsaturated FAs, PUFAs; total n3 FAs; and total n6 FAs) and FA ratios (total n3 FAs/total n6 FAs and polyene index, PI) were determined taking into account the results obtained for individual FAs. The PI was determined as the following ratio of FA concentrations: C20:5n3 + C22:6n3/C16:0.

### 2.6. Determination of TMA Content

TMA-nitrogen (TMA-N) content was analyzed spectrophotometrically (410 nm) on the basis of the picrate method in agreement with previous research [44]. For the analysis, a 5% trichloroacetic acid extract of fish muscle (10 g/25 mL) was prepared. TMA values were calculated as mg TMA-N·kg^−1^ fish muscle.

### 2.7. Statistical Analysis

Data (*n* = 3) from the different quality analyses were submitted to one-way ANOVA. Two different factors, i.e., concentration of AF extract in the glazing system and previous frozen storage time, were analyzed (Statistica version 6.0, 2001; Statsoft Inc. Tulsa, OK, USA). The least-squares difference (LSD) method was used. In all cases, a significant confidence interval at the 95% level (*p* < 0.05) was taken into account.

## 3. Results and Discussion

### 3.1. Determination of Lipid Oxidation Evolution

The peroxide content of the initial fish (i.e., 0.35 meq. active oxygen·kg^−1^ lipids) (Table 1) can be considered low and would correspond to a high-quality fish [2,45,46]. After freezing and undergoing a frozen period, a slight PV increase (*p* < 0.05) was proved in all samples. However, values were included in the 0-2-score range, which can be considered a low value for frozen seafood [2,45,46]. Comparison among the different batches did not lead to differences (*p* > 0.05) after 3 months of frozen storage. However, a lower PV was observed in fish corresponding to the L-AF batch when compared to all other batches after a 6-month storage.

TBARS values obtained were included in the 0.20–0.60-score range in all batches (Table 1). No effect (*p* > 0.05) on the TBARS content was observed after freezing and frozen storage for 3 months. However, a remarkable increase (*p* < 0.05) was detected in all batches as a result of increasing the frozen period up to a 6-month period. Comparison among batches indicated a lower average value in samples corresponding to the M-AF batch when compared to the remaining samples; this difference was found significant (*p* < 0.05) when considering the fish muscle at the end of the study. Therefore, a selective effect of the AF concentration could be concluded. As an explanation, previous research has proven that the antioxidant activity may depend on different factors, such as lipid composition, antioxidant concentration, temperature, oxygen pressure, and the presence of other antioxidants [47,48].

**Figure 1:**
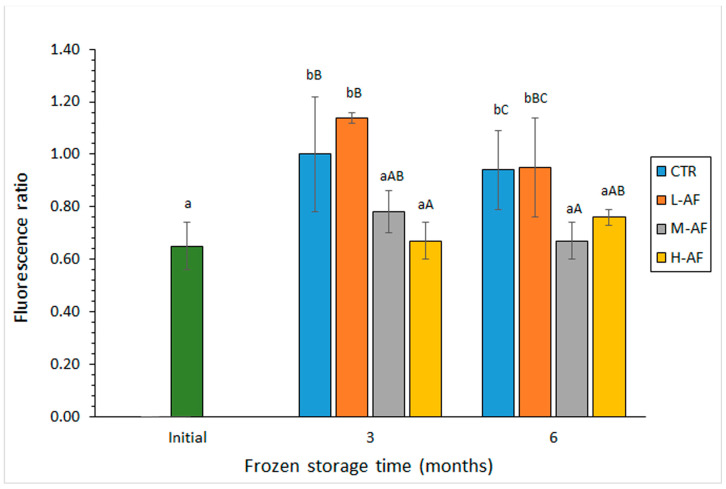
Assessment of the fluorescence ratio in frozen horse mackerel subjected to glazing including different alga flour (AF) contents. Average values of three independent determinations (*n* = 3); standard deviations are indicated by bars. Different lowercase letters denote significant differences (*p* < 0.05) with the frozen storage time; at each frozen storage time, different capital letters denote significant differences (*p* < 0.05) with the glazing condition. Glazing conditions as expressed in Table 1.

The interaction compounds (tertiary lipid oxidation compounds) resulting from reaction of primary and secondary lipid oxidation compounds with nucleophilic compounds present in the fish muscle were determined by fluorescence detection (i.e., the FR) [35,36,41]. As a result of this measurement, an average increase in the FR was observed in all batches after freezing and frozen storage for 3 months (Figure 1); this increase was found significant (*p* < 0.05) in samples corresponding to the CTR and L-AF batches. No effect (*p* > 0.05) was detected by increasing the frozen storage time up to a 6-month period in any of the batches considered. Comparison among batches indicated lower average values in fish corresponding to the H-AF batch; differences were found significant (*p* < 0.05) at both storage times by comparison to the CTR batch. Lower average values were also detected in fish corresponding to the M-AF batch when compared to their counterparts from the CTR and L-AF batches at both storage times.

Lipid oxidation is reported to be a complex deteriorative pathway since it includes the formation of different kinds of compounds, most of them unstable and susceptible to breakdown and producing lower-weight compounds, which in turn would react with nucleophilic-type molecules (free amino acids, peptides, proteins, aminated phospholipids, etc.) present in the muscle of marine species [41,47,49]. Since this study concerns frozen storage, lipid oxidation evolution should mainly be originated by the action of endogenous enzymes (i.e., peroxidases, lipoxygenases, oxidases, etc.) [1,50]. According to the present results, a general formation of primary, secondary, and tertiary lipid oxidation compounds was proved in all cases during the storage period. Nevertheless, the presence of the AF extract in the glazing system led to an inhibitory effect (TBARS content and FR).

Previous research has reported on the preservative effect of AF extract on seafood substrates. In agreement with the current results, an antioxidant effect of aqueous extracts of *Gelidium* sp. flour was reported in a model study including a heated (50 °C up to 11 days) fish muscle system [30]; in this study, the presence of the AF extracts led to a lower fluorescent compound formation and to a preservative effect on conjugated diene and triene compounds in the fish muscle.

The study of the molecules responsible for the preserving effect was not achieved in the current study. Nevertheless, taking into account the fact that a water extract of AF was employed, antioxidant activity found can be justified by the presence of hydrophilic molecules in the flour [51,52]. Previous in vitro and seafood studies have already proved the antioxidant activity of red alga constituents. Related to the present research, previous in vitro studies have proved an antioxidant behavior of water extracts from *Hypnea flagelliformis* [53], *Gracilaria verrucosa* [54], and *Gracilaria gracilis* [55]. In such studies, these effects were linked to the presence of carbohydrates, phenolic compounds (alkaloids, flavonoids), etc. Among such preservative compounds, carbohydrates have attracted a remarkable interest. Thus, polysaccharide compounds obtained from *Gracilaria corticata* [27]; *Porphyra yezoensis* [56]; and *Portieria hornemannii*, *Asparagopsis taxiformis*, *Spyridia hypnoides*, and *Centroceras clavulatum* [57] were found be responsible for the remarkable antioxidant activities during the development of in vitro studies.

Related to the present kind of extract used, different attempts have been made to analyze carbohydrate composition in different red algae species. Thus, cold-water extracts from red alga *Pterocladia capillacea* showed an enriched content of glucose, arabinose, and glucuronic acid, while hot-water extracts revealed high levels of fructose and glucuronic acid [58]. Pei et al. [28] indicated that glucose, galactose, xylose, rhamnose, glucuronic acid, and L-fucose were the major components in *Gelidium pristoides*. The presence of glucose, fucose, arabinose, galactose, and xylose was also reported by Olasehinde et al. [59] as the most abundant monosaccharides in sulfated polysaccharides from *Gelidium pristoides*.

Regarding the use of red alga extracts in seafood systems, inhibition of lipid oxidation (determination of PV and *p*-anisidine value) and preservation of astaxanthin and tocopherol levels were observed in canned salmon (*Salmo salar*) as a result of including extracts from red algae *Pyropia columbina* and *Gracilaria chilensis* in the packaging media [60]. Later on, an inhibitory effect on the FR was detected in chilled hake (*Merluccius merluccius*) by incorporating an ethanolic-aqueous extract of red alga *Gracilaria gracilis* in the icing medium [61]. A previous dipping in aqueous extracts of red algae *Gracilafia chilensis*, *Iridaea larga*, *Gelidium chilense*, *Gigartina radula*, *Gigartina chamissoi*, and *Gigartina akottsbergii* led to lower lipid oxidation development and higher retention of endogenous antioxidants (i.e., astaxanthin and tocopherols) in cooked salmon paste [62]; this behavior was attributed to the presence of preservative compounds such as polyphenols, carotenoids, phlorotannins, diterpenes, and phytosterols in alga extracts. The employment of a brown alga (*Cystoseira stricta*) extract as glazing system led to an inhibitory effect on the development of lipid oxidation (peroxide, TBA, and fluorescence indices) and higher retention of α-tocopherol in frozen (−18 °C for 9 months) Atlantic Chub mackerel (*Scomber colias*) [63].

### 3.2. Determination of Lipid Hydrolysis Evolution

The initial fish showed a low FFA level (i.e., 35.4 g FFA·kg^−1^ muscle) (Table 2) [50,64]. After freezing and frozen storage, a great increase (*p* < 0.05) in the FFA value was proved in all batches after a 3-month storage; however, no effect (*p* > 0.05) was proved by increasing the storage period up to a 6-month period. Comparison among glazing systems indicated a progressive increase (*p* < 0.05) in the FFA level in frozen fish according to the following sequence: CTR < L-AF < H-AF, M-AF. It is concluded that the employment of the two most concentrated AF extracts led to higher values (*p* < 0.05) when compared to samples corresponding to the CTR batch.

Lipid hydrolysis development in frozen fish muscle is reported to be produced as a result of release of endogenous lipases from liposomes into the muscle, which then facilitates closer proximity between enzyme and lipid substrate, i.e., high-molecular-weight lipid molecules like triacylglycerols (TAGs) and phospholipids (PLs) [1,50,64]. Formation of FFAs would increase with the frozen storage time. However, FFAs are likely to be oxidized or broken down during processing in general, as they have a greater accessibility to pro-oxidant molecules than do TAGs and PLs [65,66]; consequently, this effect would provoke a FFA content decrease. Since a remarkable increase during the current study has been produced during frozen storage, the catalytic effect of endogenous enzymes has revealed to be more important in agreement with previous studies on seafood stored under frozen conditions [3,64]. In the present study, higher FFA values (*p* < 0.05) were observed in frozen fish subjected to glazing systems including the two most concentrated AF conditions. This effect can be explained by the creation of less favorable conditions for the interaction between endogenous enzymes and TAGs and PLs in fish muscle [1,67]. It is therefore concluded that the presence of such extracts has led to a protective effect on FFA molecules. In agreement with the present results, a preservative effect was inferred on the FFA content in a heated-fish (50 °C up to 11 days) system by addition of an aqueous extract of *Gelidium* sp. flour [30]; this preservative effect showed an increase as the concentration of the AF increased.

In agreement with the results of the present study, a previous treatment with an aqueous extract from red alga *Polysiphonia fucoides* led to an enhanced FFA content in chilled minced Atlantic mackerel (*Scomber scombrus*) [68]. Similarly, the inclusion of an aqueous extract of *C. stricta* in the glazing system showed an inhibitory effect on the FFA value of frozen Atlantic Chub mackerel (*S. colias*) [63]. However, the presence of an ethanolic-aqueous extract of red alga *G. gracilis* in the icing medium did not provoke any effect on the FFA value of chilled hake (*M. merluccius*) [61].

### 3.3. Determination of the TMA Content

A low TMA value was detected in the initial fish (i.e., 0.05 mg TMA-N·kg^−1^ muscle) (Table 2), which corresponds to a high-quality fish [45]. A great increase (*p* < 0.05) was observed after freezing and frozen storage for 3 months in all batches. No additional increase (*p* > 0.05) in the TMA value was obtained by increasing the storage time up to a 6-month period. Comparison among samples did not provide significant differences (*p* > 0.05) as a result of the presence of the AF extract in the glazing system.

Endogenous enzymes and spoilage bacteria are reported to decompose food proteins and lead to nitrogen-containing amine formation [50,69]. As freezing, frozen storage, and thawing processes are involved in the current study, a volatile amine formation like TMA would likely be produced by the action of fish enzymes (i.e., proteases) on protein-derived molecules present in the frozen fish muscle [1,50]; this effect would increase with storage time [1,50]. This damage pathway would justify the present remarkable increase in the TMA content in all frozen samples. In the present study, no effect of the current AF extract on the evolution of this deteriorative mechanism in the horse mackerel muscle during the frozen storage period was proved. Therefore, a preservative effect on protein degradation was not concluded by including the AF extract in the glazing medium.

Contrary to the present results, an inhibitory effect on TMA formation was detected in Atlantic Chub mackerel (*S. colias*) by including an aqueous extract of *C. stricta* in the glazing medium employed [63]. The inclusion of extracts obtained from red algae in the icing medium has proved to have an inhibitory effect on the TMA value during the chilling storage of fish. This is the case with methanolic *G. verrucosa* extracts in chilled Indian mackerel (*R. kanagurta*) [70], ethanolic-aqueous extracts of *G. gracilis* in chilled hake (*M. merluccius*) [61], and aqueous extracts of *Gelidium* sp. flour in chilled Atlantic mackerel (*S. scombrus*) [29].

### 3.4. Determination of the FA Profile

The FA profile of the initial fish revealed the following composition for individual FAs (g·100 g^−1^ total FAs): 3.11 ± 0.52 (C14:0), 0.56 ± 0.10 (C15:0), 21.07 ± 0.66 (C16:0), 3.52 ± 1.69 (C16:1n7), 0.86 ± 0.16 (C17:0), 6.79 ± 0.60 (C18:0), 15.94 ± 5.25 (C18:1n9), 3.08 ± 0.45 (C18:1n7), 1.42 ± 0.22 (C18:2n6), 1.26 ± 0.23 (C20:1n9), 0.29 ± 0.09 (C20:2n6), 1.51 ± 0.35 (C20:4n6), 0.25 ± 0.02 (C22:1n9), 8.05 ± 0.88 (C20:5n3), 0.35 ± 0.05 (C22:4n6), 0.66 ± 0.25 (C24:1n9), 2.97 ± 0.57 (C22:5n3), and 26.29 ± 7.04 (C22:6n3). The following composition of FA groups was obtained (g·100 g^−1^ total FAs): 32.47 ± 1.29 (STFAs), 25.71 ± 7.29 (MUFAs), 41.82 ± 8.35 (PUFAs), 38.19 ± 7.77 (total n3 PUFAs), and 3.63 ± 0.66 (total n6 PUFAs). Additionally, the following FA ratios were obtained: 10.50 ± 1.05 (total n3/total n6 ratio) and 1.68 ± 0.39 (PI).

To better analyze changes produced in the FA profile in the present research, the analysis and discussion of the FA results will be focused on the content of FA groups (i.e., STFAs, MUFAs, and PUFAs) and FA ratios (n3/n6 and PI).

A content increase (*p* < 0.05) in the STFA value was detected in all samples after freezing and a 3-month period of frozen storage (Table 3); no effect (*p* > 0.05) was proved in any batch if a subsequent increase in the storage period to 6 months was achieved. An average content decrease was detected in all batches for the PUFA group after freezing and a 6-month period of frozen storage (Table 3); nevertheless, differences were not found significant (*p* > 0.05) for any of the batches considered. Regarding the MUFA group, a definite tendency could not be proved with freezing and frozen storage (Table 3).

Regarding the effect of the AF extract included in the glazing system, differences found were very scarce (*p* > 0.05), and a definite trend could not be proved. A higher MUFA value (*p* < 0.05) was observed after a 3-month period in samples corresponding to the L-AF and M-AF batches than in their counterpart samples from the CTR and H-AF batches. For the same frozen storage period, fish corresponding to the CTR and H-AF batches provided higher PUFA levels (*p* < 0.05) than their counterpart samples corresponding to the other two batches.

Great attention has been given to the level of n3 PUFAs in regards to their valuable health benefits [71,72]. Furthermore, a remarkable interest has been accorded to the total n3/total n6 FA ratio [73,74]. Thus, recent research has demonstrated that Western citizens do not consume appropriate quantities of n3 FAs in their diets through natural sources. In order to diminish health concerns such as inflammatory, cardiovascular, and neurological disorders, the European Nutritional Society proposed that a human diet with an n3/n6 ratio of 1:5 or higher would be necessary to acquire health benefits [75]. Furthermore, the World Health Organization (WHO) indicated that this ratio should not be below 1:10 in the human diet [76].

In the present study, this FA ratio showed values included in all cases in the 10.31–11.73 range (Table 4). Therefore, it can be concluded that all kinds of samples include a highly healthy n3/n6 ratio. No effect (*p* > 0.05) of the freezing process and the frozen storage period could be proved. Neither was there an effect (*p* > 0.05) as a result of including the AF extract in the glazing system. It can be inferred that both FA groups (i.e., n3 and n6 FA series) were not differentially affected by the effects analyzed in the current study.

Regarding lipid oxidation determination, a great interest has been given to the PI as supplying complementary information regarding the development of this damage pathway in food in general. Notably, this index can afford knowledge on the possible changes of PUFA content (i.e., C20:5n3 and C22:6n3 FAs) during different processes of marine species [46,62] and provide a valuable tool regarding the nutritional properties [77,78].

In the present work, a general decrease in the average PI was observed after the freezing step and a 3-month period of frozen storage (Table 4); then, a subsequent average decrease was observed by considering a longer frozen storage period (i.e., 6 months). Differences between initial fish and samples stored for 6 months were significant (*p* < 0.05) in all batches except for fish corresponding to the H-AF batch. Higher average values were detected in fish corresponding to the M-AF and H-AF batches when compared to their counterparts from the CTR batch; differences were found significant (*p* < 0.05) when taking into account samples including the most concentrated AF extract in the glazing system.

To the best of our knowledge, no previous studies have described the effect of *Gelidium* sp. flour and macroalgae flour in general on the FA profile of seafood. Regarding the employment of red alga extracts in seafood systems, retention of the PI value was obtained in canned salmon (*S. salar*) as a result of including aqueous extracts of *P. columbina* and *G. chilensis* in the packaging medium [60]. A preservative effect on the PI was proved in frozen Atlantic Chub mackerel (*S. colias*) as a result of including an aqueous solution of alga *C. stricta* in the glazing medium [63].

## 4. Conclusions

The antioxidant properties of *Gelidium* sp. flour were analyzed and proved in a frozen-fish system. The presence of the aqueous AF extract in the glazing medium led to a lower (*p* < 0.05) TBARS and fluorescent compound formation and to higher (*p* < 0.05) PI values in frozen fish muscle. Additionally, a preservative effect on FFAs was detected in AF-treated fish. In general, preservative effects were found to be higher in frozen fish corresponding to the medium concentration of the AF extract tested in the glazing system. No effect (*p* > 0.05) of the AF-glazing treatment was implied for the TMA formation and the total n3/total n6 FA ratio.

The current results show the potential of *Gelidium* sp. flour as a natural source of preservative hydrophilic compounds susceptible to enhance the quality of frozen horse mackerel. This work opens the way to a valuable use of *Gelidium* sp. flour for the quality retention of frozen horse mackerel, its potential shelf-life extension, and its widespread commercial distribution. To the best of our knowledge, this study would provide a first and novel approach to the employment of this red alga waste in frozen seafood. According to the abundance of *Gelidium* sp., this strategy agrees with present global interests in sustainable food technology and the search for new practical tools that take into account natural and underutilized sources of antioxidant compounds. Additionally, future research ought to involve detailed analysis of active preservative compounds present in the current AF extract.

## Figures and Tables

**Table 1:** Determination * of peroxide value (PV) and thiobarbituric acid index (TBA-i) in frozen horse mackerel subjected to glazing including different alga flour (AF) contents **.

Quality Index	Storage Time (Months)	Glazing System			
		CTR	L-AF	M-AF	H-AF
PV (meq. active oxygen·kg^−1^ lipids)	Initial	0.35 a (0.12)	0.35 a (0.12)	0.35 a (0.12)	0.35 a (0.12)
	3	1.06 bA (0.36)	0.71 abA (0.25)	0.92 bA (0.18)	0.89 bA (0.04)
	6	1.54 bB (0.36)	0.83 bA (0.33)	1.73 cB (0.05)	1.48 cB (0.10)
TBA-i (mg malondialdehyde·kg^−1^ muscle)	Initial	0.20 a (0.10)	0.20 a (0.10)	0.20 a (0.10)	0.20 a (0.10)
	3	0.21 aAB (0.12)	0.20 aAB (0.07)	0.19 aA (0.04)	0.34 aB (0.08)
	6	0.60 bB (0.11)	0.55 bB (0.12)	0.35 bA (0.03)	0.57 bB (0.08)

* Average values of three independent determinations (*n* = 3); standard deviations are expressed in brackets. In each column, different lowercase letters indicate significant differences (*p* < 0.05) with the frozen storage time; in each row and for each index, different capital letters denote significant differences (*p* < 0.05) with the glazing condition. ** Glazing conditions: CTR (control; glazing prepared without AF extract); L-AF, M-AF, and H-AF correspond to low, medium, and high concentrations of AF extracts in the glazing system, respectively).

**Table 2:** Determination * of free fatty acid (FFA) and trimethylamine (TMA) values in frozen horse mackerel subjected to glazing including different alga flour (AF) contents **.

Quality Index	Storage Time (Months)	Glazing System			
		CTR	L-AF	M-AF	H-AF
FFAs (mg·kg^−1^ muscle)	Initial	35.4 a (6.5)	35.4 a (6.5)	35.4 a (6.5)	35.4 a (6.5)
	3	1043.3 bA (16.2)	1355.5 bB (24.5)	1434.1 bC (36.6)	1424.5 bC (32.7)
	6	1088.2 bA (20.3)	1283.3 bB (30.9)	1458.1 bC (21.5)	1435.9 bC (29.6)
TMA (mg TMA-N·kg^−1^ muscle)	Initial	0.05 a (0.01)	0.05 a (0.01)	0.05 a (0.01)	0.05 a (0.01)
	3	0.76 bA (0.16)	0.73 bA (0.16)	0.86 bA (0.17)	0.99 bA (0.19)
	6	0.79 bA (0.14)	0.67 bA (0.20)	0.62 bA (0.02)	0.74 bA (0.08)

* Average values of three independent determinations (*n* = 3); standard deviations are indicated in brackets. In each column, different lowercase letters denote significant differences (*p* < 0.05) with the frozen storage time; in each row and for each index, different capital letters denote significant differences (*p* < 0.05) with the glazing condition. ** Glazing conditions as expressed in Table 1.

**Table 3:** Content of fatty acid (FA) groups (g·100 g^−1^ total FAs) * in frozen horse mackerel subjected to glazing including different alga flour (AF) contents **.

FA Group	Storage Time (Months)	Glazing System			
		CTR	L-AF	M-AF	H-AF
Saturated FAs	Initial	32.47 a (1.29)	32.47 a (1.29)	32.47 a (1.29)	32.47 a (1.29)
	3	35.33 bA (0.61)	35.29 bA (0.24)	35.94 bA (0.77)	35.28 bA (1.34)
	6	35.10 bA (0.52)	34.99 bA (0.57)	35.11 bA (0.93)	35.01 bA (0.82)
Monounsaturated FAs	Initial	25.71 a (7.29)	25.71 a (7.29)	25.71 a (7.29)	25.71 a (7.29)
	3	22.86 aA (4.31)	34.59 aB (1.94)	32.77 aB (1.07)	23.87 aA (4.23)
	6	27.01 aA (6.08)	28.07 aA (7.35)	31.64 aA (2.50)	27.58 aA (5.86)
Polyunsaturated FAs	Initial	41.82 a (8.35)	41.82 a (8.35)	41.82 a (8.35)	41.82 a (8.35)
	3	41.81 aB (4.78)	30.12 aA (1.83)	31.30 aA (1.85)	40.84 aB (5.25)
	6	37.89 aA (6.46)	36.94 aA (7.57)	33.25 aA (1.57)	37.40 aA (6.45)

* Average values of three independent determinations (*n* = 3); standard deviations are indicated in brackets. In each column, different lowercase letters denote significant differences (*p* < 0.05) with the frozen storage time; in each row and for each FA group, different capital letters denote significant differences (*p* < 0.05) with the glazing condition. ** Glazing conditions as expressed in Table 1.

**Table 4:** Fatty acid (FA) ratios * in frozen horse mackerel subjected to glazing including different alga flour (AF) contents **.

FA Ratio	Storage Time (Months)	Glazing System			
		CTR	L-AF	M-AF	H-AF
Total n3 FAs/total n6 FAs	Initial	10.50 a (1.05)	10.50 a (1.05)	10.50 a (1.05)	10.50 a (1.05)
	3	10.85 aA (0.28)	10.46 aA (0.46)	10.31 aA (0.48)	11.39 aA (0.88)
	6	10.06 aA (1.07)	10.73 aA (0.58)	10.03 aA (0.23)	10.37 aA (0.61)
Polyene index	Initial	1.68 b (0.39)	1.68 b (0.39)	1.68 b (0.39)	1.68 a (0.39)
	3	1.24 bA (0.07)	1.21 abA (0.15)	1.35 abAB (0.23)	1.50 aB (0.12)
	6	1.08 aA (0.04)	1.09 aAB (0.08)	1.19 aAB (0.03)	1.36 aB (0.19)

* Average values of three independent determinations (*n* = 3); standard deviations are indicated in brackets. In each column, different lowercase letters denote significant differences (*p* < 0.05) with the frozen storage time; in each row and for each index, different capital letters denote significant differences (*p* < 0.05) with the glazing condition. ** Glazing conditions as expressed in Table 1.

## Data Availability

The original contributions presented in the study are included in the article, further inquiries can be directed to the corresponding author.

## Abbreviations

AF	alga flour
CTR	control
FAMEs	fatty acid methyl esters
FAs	fatty acids
FFAs	free fatty acids
FR	fluorescence ratio
H-AF	high-concentrated alga flour
L-AF	low-concentrated alga flour
M-AF	medium-concentrated alga flour
MUFAs	monounsaturated fatty acids
PI	polyene index
PLs	phospholipids
PUFAs	polyunsaturated fatty acids
PV	peroxide value
RF	relative fluorescence
STFAs	saturated fatty acids
TAGs	triacylglycerols
TBA	thiobarbituric acid
TBA-i	thiobarbituric acid index
TBARSs	thiobarbituric acid reactive substances
TMA	trimethylamine
TMA-N	trimethylamine-nitrogen
